# Supplementary material for: Pilot Study for Immunogenicity of SARS-CoV-2 Vaccine with Seasonal Influenza and Pertussis Vaccines in Pregnant Women
Source: Vaccines (Basel). 2023 Jan 3;11(1):119. doi: 10.3390/vaccines11010119 (PMC9860598; doi:10.3390/vaccines11010119)
Supplement: Supplementary file 1 [file vaccines-11-00119-s001.zip › vaccines-2146723.pdf]

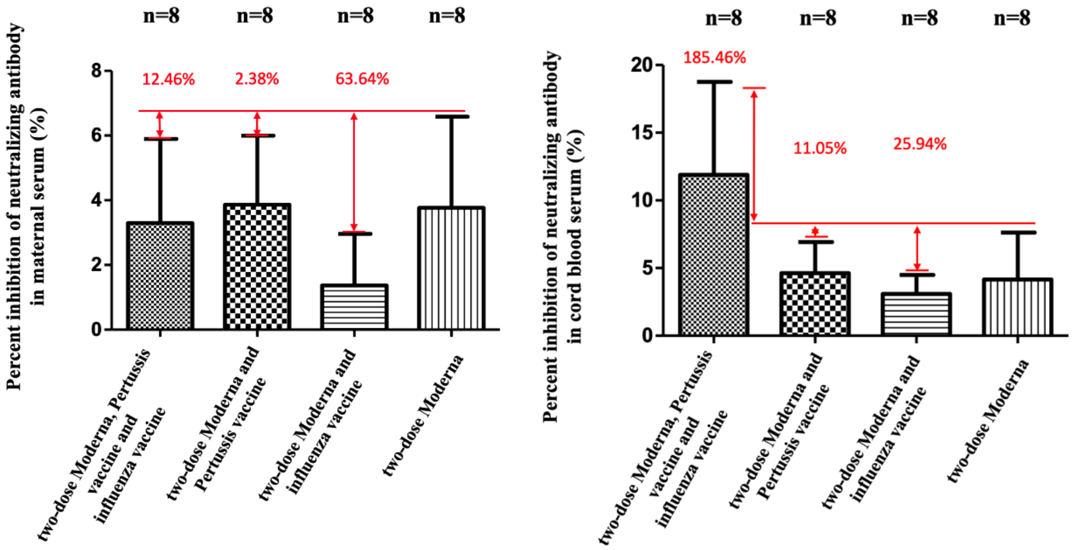

Figure S1. The percentage of inhibition of neutralizing antibody (%) against the Omicron variant in maternal and cord blood.

Table S1. Concentration(Unit) of Anti-SARS-CoV-2 total antibody in maternal blood.

**3 Moderna + Tdap + Flu (n=7)**	>2500	>2500	>2500	>2500	>2500	1688	>2500	
**2AZ+1 Moderna + Tdap +Flu (n=6)**	>2500	>2500	>2500	1802	1095	2137		
**2 Moderna + Tdap + Flu (n=8)**	>2500	984.9	1900	771.6	1079	>2500	1177	1095

Table S2. Concentration(Unit) of Anti-SARS-CoV-2 total antibody in cord blood.

**3 Moderna + Tdap + Flu(n=7)**	>2500	2346	>2500	>2500	>2500	2495	>2500	
**2AZ+1 Moderna + Tdap +Flu (n=6)**	>2500	>2500	>2500	>2500	2417	>2500		
**2 Moderna + Tdap + Flu (n=8)**	>2500	>2500	>2500	825.6	1519	>2500	1817	1612
